# Effects of Iodine Status and Vitamin A Level on Blood Pressure, Blood Glucose, and Blood Lipid Levels in Chinese Adults: A Cross-Sectional Study

**DOI:** 10.3390/nu17243948

**Published:** 2025-12-17

**Authors:** Jingtao Zhao, Manman Chen, Yang Peng, Keyu Han, Qu Lu, Bin Dong

**Affiliations:** 1 School of Business, Jiangxi Institute of Fashion Technology, Nanchang 330201, China; zhaojingtao@jift.edu.cn; 2 School of Population Medicine and Public Health, Chinese Academy of Medical Sciences and Peking Union Medical College, Beijing 100730, China; chenmm@pumc.edu.cn; 3 Faculty of Health, Medicine and Behavioural Sciences, The University of Queensland, Brisbane, QLD 4072, Australia; yang.peng@uq.net.au; 4 School of Health Policy and Management, Chinese Academy of Medical Sciences and Peking Union Medical College, Beijing 100730, China; 13515231601@163.com (K.H.); qulu167@163.com (Q.L.); 5 Institute of Child and Adolescent Health, School of Public Health, Peking University Health Science Center, No. 38 Xueyuan Road, Haidian District, Beijing 100191, China

**Keywords:** iodine deficiency, blood pressure, blood glucose, blood lipid, vitamin A levels

## Abstract

Background: Iodine deficiency remains a significant public health concern worldwide and may contribute to metabolic disorders beyond thyroid dysfunction. Emerging evidence suggests that nutritional factors, such as vitamin A, may influence the health effects of iodine deficiency, yet population-based evidence remains limited. This study aimed to investigate the associations between iodine deficiency and cardiometabolic risk factors (blood pressure, glucose, and lipids) and to explore whether these associations are different between adults with different vitamin A levels. Methods: A total of 4723 adults (1895 males and 2828 females) were included in this cross-sectional study. Participants were categorized based on iodine status and serum vitamin A levels. Demographic, anthropometric, and biochemical indicators were assessed through standardized examinations. Multivariable linear and logistic regression models were used to evaluate the associations between iodine deficiency and continuous (systolic blood pressure [SBP], diastolic blood pressure [DBP], fasting blood glucose [FBG], total cholesterol [TC], high-density lipoprotein cholesterol [HDL-C], low-density lipoprotein cholesterol [LDL-C], triglycerides [TGs]) and binary outcomes (hypertension, hyperglycemia, and dyslipidemia), with stratified analyses by gender, age, and vitamin A status. Results: Iodine deficiency was significantly associated with higher SBP (β = 2.89, 95% confidence interval [CI]: 2.00–3.77), DBP (β = 1.08, 0.55–1.60), FBG (β = 0.06, 0.01–0.12) and TC (β = 0.05, 0.00–0.10). The odds of hypertension (odds ratio [OR] = 1.41, 1.23–1.63) and hyperglycemia (OR = 1.39, 1.17–1.65) were also increased. Stratified analyses indicated that these associations were more pronounced among participants with vitamin A deficiency than those with sufficient vitamin A. In this subgroup, iodine deficiency was positively associated with FBG (β = 0.14, 0.03–0.25), TC (β = 0.08, 0.00–0.15), and hyperglycemia (OR = 1.35, 1.04–1.76). Conclusions: The findings suggest that the association of iodine deficiency with adverse cardiometabolic risk factors may be stronger in individuals with concurrent vitamin A deficiency. This highlights the potential value of integrated nutritional assessments and supports the need for longitudinal studies to confirm these interactions and assess the effects of combined micronutrient supplementation.

## 1. Introduction

Iodine, an essential trace element in human nutrition, exhibits antioxidant properties and has been associated with protective effects against inflammatory states and carcinogenesis [1,2]. Urinary iodine level is regarded as the most sensitive and reliable indicator for assessing population iodine intake, as approximately 90% of the ingested iodine is excreted through the kidneys [3]. The World Health Organization recommends a daily iodine intake of 0.15 milligrams for adults [4]. Iodine deficiency in adults may lead to secondary hypothyroidism, resulting in apathetic emotions and reduced work efficiency, and is typically indicated by a urinary iodine level below 100 μg/L [5,6]. Research has shown that iodine deficiency is associated with adverse health outcomes [7,8,9,10], and related evidence highlights the urgent need for dietary adjustments and early detection strategies to manage and mitigate the health risks associated with this overlooked condition.

Studies have illustrated that elevated thyrotropin (TSH) levels have been associated with dyslipidemia [11], increased body mass index (BMI) [12,13], and higher mortality from coronary artery disease [14]. In mild to moderate iodine deficiency, circulating thyroxine (T4) levels decrease due to impaired thyroid hormone synthesis, triggering increased TSH secretion via feedback regulation of the hypothalamic-pituitary-thyroid axis. This stimulates thyroid gland hyperplasia and enhances iodine uptake [14]. However, when this compensatory state becomes chronic, the persistently elevated TSH exerts direct adverse effects [15]. It promotes hepatic lipogenesis and reduces lipid clearance, contributing to dyslipidemia [11]. Furthermore, TSH can stimulate preadipocyte differentiation and lead to weight gain [12], explaining the link with increased BMI. Thus, iodine deficiency has been acknowledged as an important risk factor for metabolic and cardiovascular diseases.

The role of micronutrient interactions in modulating health outcomes is increasingly recognized [16,17,18]. Vitamin A is essential for a wide range of physiological functions, especially metabolism [19]. Studies have demonstrated that adequate vitamin A intake is significantly associated with a lower risk of all-cause mortality and cardiovascular disease and related mortality [20,21,22]. Furthermore, a randomized, double-blind, placebo-controlled trial demonstrated that vitamin A supplementation is associated with reduced levels of obesity-related Th17-Treg cytokines in women, contributing to immune modulation and improvement [23].

Despite the established individual roles of iodine and vitamin A in metabolic health, their potential interaction at the population level remains poorly characterized. The concurrent presence of these two micronutrient deficiencies may impose a compounded metabolic burden. Therefore, this cross-sectional study among 4723 Chinese adult participants aimed to investigate the associations between iodine deficiency and levels of blood pressure, blood glucose, and blood lipids, and to explore whether these associations are different in adults with different vitamin A status. Our findings may help provide population-based evidence for guiding future longitudinal and interventional research.

## 2. Materials and Methods

### 2.1. Study Design and Participants

**Figure 1:**
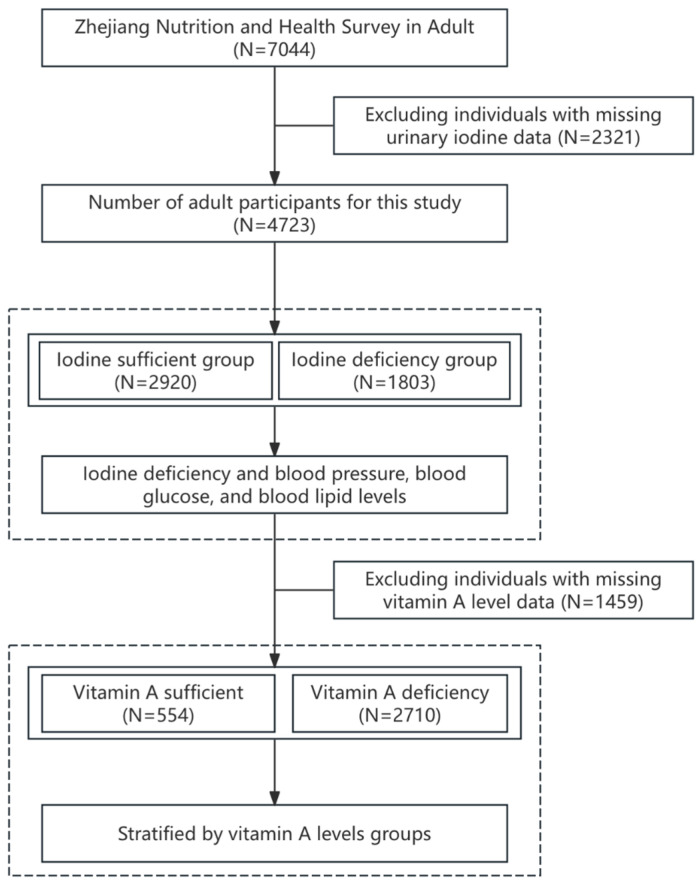
Flowchart of the study participants.

A cross-sectional survey was carried out in 2022 to obtain a representative sample of adults from Zhejiang Province, China, using a multi-stage stratified random sampling scheme. Zhejiang comprises eleven prefectural cities and 90 counties/districts [24,25]. In the first stage, two counties (or districts) were randomly selected from each city (22 in total). In the second stage, three towns (or streets) were randomly selected from each chosen county, yielding 66 towns/streets. Finally, one community was randomly sampled within each selected town or street. Individuals who had been exposed within the previous six months to iodinated contrast agents (for procedures such as coronary angiography or endoscopic retrograde cholangiopancreatography), who had used amiodarone, or who had severe psychiatric disorders or dementia were excluded a priori. The study enrolled 4723 adults aged 18 years or older. Minors were excluded because the study focused on adult cardiometabolic outcomes and because of the distinct physiological and ethical considerations for individuals under 18 years of age (Figure 1).

The study protocol was approved by the Ethical Committee of the Zhejiang Provincial Center for Disease Control and Prevention (CDC) (approval code: 2022-018-01; approval date: 10 May 2022). Written informed consent was obtained from all participants.

### 2.2. Measurements and Classifications

**Urinary iodine concentration and definition of iodine deficiency.** Spot urine samples were collected and analyzed for urinary iodine concentration (UIC) using the arsenic (III)–cerium (IV) (As^3+^–Ce^4+^) catalytic spectrophotometric method. Following World Health Organization (WHO) criteria (https://www.who.int/data/nutrition/nlis/info/iodine-deficiency, accessed on 27 November 2025), iodine deficiency was defined as UIC < 100 μg/L, which corresponds to the threshold for insufficient iodine intake in the general population. The UIC is considered the main indicator of iodine status because it is a sensitive marker of current iodine intake. For analytical purposes, iodine deficiency was treated as a binary exposure (iodine deficiency, UIC < 100 μg/L; iodine sufficiency, UIC 100–300 μg/L).

**Blood pressure, blood glucose, and blood lipid levels.** Trained staff measured blood pressure following a standardized protocol: participants rested quietly for at least five minutes while seated with back support and feet flat on the floor, and an appropriately sized cuff was applied to the bare upper arm at heart level. At least two readings were taken 1–2 min apart using a validated automated device; the mean of the readings was used for analysis. According to the 2023 European Society of Hypertension (ESH) guideline update, hypertension was defined in this study as systolic blood pressure (SBP) ≥ 130 mmHg or diastolic blood pressure (DBP) ≥ 80 mmHg, or current use of antihypertensive medication [26]. Fasting plasma glucose (FPG) was measured from venous blood collected after an overnight fast of at least eight hours; hyperglycemia was defined as FPG ≥ 7.0 mmol/L (126 mg/dL) or current use of glucose-lowering treatment. Serum lipids measured included total cholesterol (TC), high-density lipoprotein cholesterol (HDL-C), low-density lipoprotein cholesterol (LDL-C), and triglycerides (TGs). Binary dyslipidemia outcomes were defined according to routine clinical cutoffs and/or current use of lipid-lowering therapy; specifically, low HDL-C was defined as HDL-C < 1.03 mmol/L in men and <1.30 mmol/L in women. Continuous lipid values were analyzed as measured.

**Vitamin A and vitamin D.** Venous blood samples were processed with separation gel and the supernatant was used for biochemical assays. Serum vitamin A (retinol) and vitamin D concentrations were quantified by liquid chromatography–tandem mass spectrometry (LC-MS/MS). Subclinical vitamin A deficiency was defined according to WHO criteria as serum retinol <0.70 μmol/L (https://www.who.int/data/nutrition/nlis/info/vitamin-a-deficiency, accessed on 23 October 2025). Vitamin D was included as a covariate and measured by LC-MS/MS using the same platform.

### 2.3. Other Covariates

All participants received a standardized physical examination conducted by trained public health physicians. Height and weight were measured to calculate BMI (kg/m^2^). Information on smoking (current daily, occasional, former, never), dietary habits (frequency of seafood, pickled foods, vegetable soup consumption, and other patterns), and typical sleep duration (≥8 h or <8 h) was collected using a structured questionnaire administered by trained interviewers. Demographic variables, medication use, and other health history items were also recorded. Participants with missing data on the primary exposure or outcome were excluded, while multiple imputation was used to handle missing covariates.

### 2.4. Statistical Analysis

Descriptive analyses were first conducted to characterize the study population. Continuous variables were summarized as means and standard deviations for normally distributed data or as medians and interquartile ranges for skewed distributions, while categorical variables were expressed as frequencies and percentages. Group differences between participants with and without iodine deficiency were examined using the independent-samples *t*-test or Mann–Whitney U test for continuous variables and the chi-square test for categorical variables.

Multivariable linear regression models were used to examine associations between iodine deficiency and continuous outcomes (SBP, DBP, FBG, TC, HDL-C, LDL-C, and TGs), with regression coefficients (β) and 95% confidence intervals (CI) reported. Logistic regression models were applied to assess associations with binary outcomes, including hypertension, hyperglycemia, and dyslipidemia, and the results were expressed as odds ratios (OR) with 95% CI. Age, gender, BMI, smoking status, dietary habits, sleeping time, and vitamin D levels were included as covariates to address potential confounding from baseline characteristics.

Effect modification by vitamin A status was examined by adding an interaction term between iodine deficiency and vitamin A deficiency in the adjusted models. Stratified analyses were performed by vitamin A status. Further stratified analyses were conducted by gender and age group (18–50 years and >50 years) to explore heterogeneity in associations.

To address potential bias from group imbalance and to evaluate the robustness of stratified analyses, participants were also categorized into lower vitamin A and higher vitamin A groups based on the median serum vitamin A concentration (0.52 μmol/L).

All tests were two-sided, and a *p*-value < 0.05 was considered statistically significant. Statistical analyses were conducted using R software (version 4.3.2; R Core Team, Vienna, Austria).

## 3. Results

### 3.1. Participant Characteristics

This study included a total of 4723 participants (1895 males and 2828 females), as presented in Table 1. Of these, 2174 individuals (46.03%) were aged 18–50 years, and 2549 individuals (53.97%) were older than 50 years. A total of 3264 participants (69.1%) had validated records of vitamin A levels (Table S1). In comparison with the original cohort, participants with recorded vitamin A levels were, on average, slightly younger. However, the differences in the distributions of key demographic subgroups were generally below 5%, suggesting that the potential for selection bias is relatively minor. Significant differences in basic demographic and clinical characteristics were observed between the vitamin A sufficient and vitamin A deficiency groups (*p* < 0.05). The vitamin A sufficient group demonstrated significantly higher mean values for height, weight, BMI, SBP, DBP, FBG, TC, and TGs compared to the vitamin A deficiency group (all *p* < 0.001). No statistically significant difference was found for LDL-C between the two groups (*p* = 0.075). Meanwhile, the vitamin A sufficient group had a significantly higher prevalence of hypertension, hyperglycemia, high TC, low HDL-C, high LDL-C, and high TGs compared to the deficiency group (all *p* < 0.05).

### 3.2. The Distribution of Iodine Deficiency Across Different Gender and Age Groups

**Figure 2:**
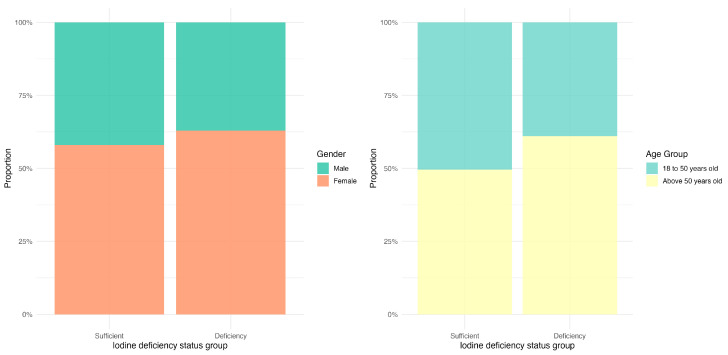
Bar chart of the proportion of gender and age groups among participants with different iodine deficiency status.

Figure 2 illustrates the distribution of iodine status across different gender and age groups. The proportion of females was notably higher than that of males in both iodine status categories. In the iodine sufficient group, females constituted 57.8% (*n* = 1693), compared to 42.0% (*n* = 1227) for males. In the iodine deficiency group, females accounted for 63.0% (*n* = 1135), whereas males represented 37.0% (*n* = 668). Regarding age distribution, individuals aged 18–50 years constituted a slightly larger share of the iodine sufficient group, representing 50.4% (*n* = 1472), compared to 49.6% (*n* = 1448) for participants older than 50 years. In contrast, the iodine deficiency group was predominantly older, with participants above 50 years accounting for 61.1% (*n* = 1101), while those aged 18–50 years represented 38.9% (*n* = 702).

### 3.3. Iodine Deficiency and Blood Pressure, Blood Glucose, and Blood Lipid Levels

As shown in Table 2, iodine deficiency was positively associated with higher SBP (β = 2.89, 95% CI: 2.00–3.77, *p* < 0.001), DBP (β = 1.08, 95% CI: 0.55–1.60, *p* < 0.001), FBG (β = 0.06, 95% CI: 0.01–0.12, *p* = 0.031), and TC (β = 0.05, 95% CI: 0.00–0.10, *p* = 0.033) in adjusted models. Iodine deficiency was also associated with increased odds of hypertension (OR = 1.41, 95% CI: 1.23–1.63, *p* < 0.001) and hyperglycemia (OR = 1.39, 95% CI: 1.17–1.65, *p* < 0.001). No significant associations were found with other lipid parameters (HDL-C, LDL-C, and TGs).

Stratified analyses revealed differential associations by gender and age (Tables S2 and S3). The association with SBP was significant in both males (β = 1.82, 95% CI: 0.28–3.36, *p* = 0.021) and females (β = 4.48, 95% CI: 2.64–6.33, *p* < 0.001), while the association with FBG was significant only in males (β = 0.26, 95% CI: 0.09–0.44, *p* = 0.003). In females, significant associations were observed for TC (β = 0.08, 95% CI: 0.01–0.16, *p* = 0.034) and hypertension risk (OR = 1.73, 95% CI: 1.44–2.09, *p* < 0.001). By age, significant associations with SBP, hypertension, and hyperglycemia were confined to participants >50 years, with no significant associations observed in the 18–50 years group.

### 3.4. Stratified by Vitamin A Levels Groups

**Figure 3:**
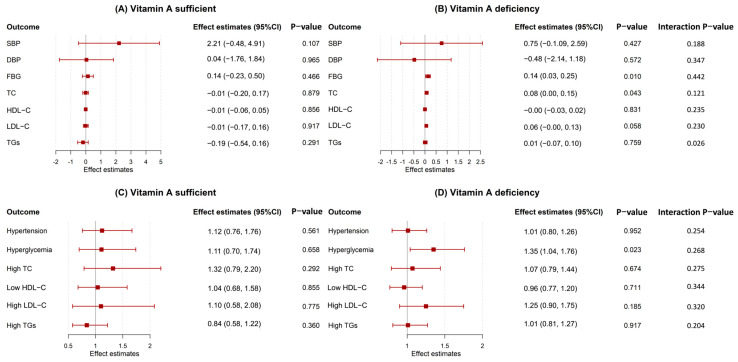
Association between iodine deficiency and blood pressure, blood glucose, and blood lipid levels, stratified by vitamin A levels. (**A**) Results for continuous variables in the vitamin A sufficient group; (**B**) Results for continuous variables in the vitamin A deficiency group; (**C**) Results for binary variables in the vitamin A sufficient group; (**D**) Results for binary variables in the vitamin A deficiency group. Note: Results are presented as numerical changes for continuous outcomes and odds ratios (ORs) for binary outcomes and their 95% confidence intervals (CIs). Squares represent effect estimates, and line segments indicate 95% CIs. Age, gender, BMI, smoking status, dietary habits, sleeping time, and vitamin D levels were adjusted. SBP, systolic blood pressure; DBP, diastolic blood pressure; FBG, fasting blood glucose; TC, total cholesterol; HDL-C, high-density lipoprotein cholesterol; LDL-C, elevated low-density lipoprotein cholesterol; TGs, triglycerides.

As shown in Figure 3, the associations between iodine deficiency and continuous levels of FBG and TC, as well as the odds of hyperglycemia, showed differences between vitamin A levels groups, with positive associations observed exclusively among participants with vitamin A deficiency. Among participants with vitamin A deficiency, iodine deficiency was associated with higher FBG (β = 0.14, 95% CI: 0.03–0.25, *p* = 0.010), TC (β = 0.08, 95% CI: 0.00–0.15, *p* = 0.043), and increased odds of hyperglycemia (OR = 1.35, 95% CI: 1.04–1.76, *p* = 0.023). In contrast, no significant associations were observed in the sufficient vitamin A group for any outcomes.

Further stratification by both vitamin A status and demographic factors revealed limited significant associations (Tables S4 and S5). A positive association between iodine deficiency and SBP was observed only in vitamin A-deficient males (β = 0.26, 95% CI: 0.04–0.47, *p* = 0.019) and in the vitamin A-deficient, >50 years age group (β = 0.20, 95% CI: 0.02–0.38, *p* = 0.031). All other cardiovascular and lipid outcomes were non-significant across the remaining strata.

The sensitivity analyses using median-based vitamin A categorization yielded consistent results (Table S6). The associations of iodine deficiency with FBG, TC, and hyperglycemia remained more pronounced in the lower vitamin A group, supporting the primary finding of effect modification, although the effect estimates were attenuated.

## 4. Discussion

In this population-based cross-sectional study of 4723 adults from Zhejiang Province, we observed that iodine deficiency was independently associated with higher SBP and DBP, modestly elevated FBG and TC, and greater odds of hypertension and hyperglycemia. A key and novel observation was that these associations were modified by vitamin A status, being more pronounced among participants with lower vitamin A levels, particularly for FBG, TC, and hyperglycemia, after controlling for potential confounding of age, sex, and other covariates. Taken together, the results suggest that iodine deficiency may contribute to an adverse cardiometabolic profile and that inadequate vitamin A status may modify these associations. This potential interaction highlights an area for public health nutrition attention.

This study indicates that urinary iodine deficiency is more prevalent among females than males. This finding is consistent with existing studies [27,28], which typically attribute this gender difference to multiple factors. Firstly, females have distinct physiological needs, such as a significant increase in iodine requirements during pregnancy and lactation [5,7]. Secondly, hormonal differences between females and males may play a role. Due to the influence of estrogen on the regulation of thyroid function and iodine metabolism, females may be more susceptible to iodine deficiency [29]. A study found that females had a 5- to 20-fold increased risk of thyroid-related diseases compared with males [27]. Additionally, this study reveals that iodine deficiency is more pronounced among individuals over 50 years of age. This result can be explained by age-related physiological changes [30]. Older adults often experience alterations in dietary habits, potentially leading to decreased consumption of iodine-rich foods [31]. Moreover, the gradual process of aging has a considerable impact on the composition of the intestinal microbiome, including the efficiency of iodine absorption [28]. The coexistence of multiple chronic conditions and frequent medication use in older groups can further impair iodine uptake and metabolism, contributing to a bidirectional cycle of worsening health outcomes [32,33,34,35]. These demographic features underscore the significance of focused surveillance of iodine nutritional status in females and older adults.

This study demonstrates that iodine deficiency is associated with increased odds of hypertension and hyperglycemia. A cohort study found that TSH was positively and linearly correlated with the mortality rate of coronary heart disease in females [13]. Previous studies have also found that iodine deficiency leads to an increase in TSH levels, which may be independently associated with adverse cardiovascular outcomes through their influence on serum lipid profiles, suggesting potential subclinical metabolic implications [11,36,37]. However, some studies have revealed that iodine supplementation did not reduce FPG [14,38]. This finding contrasts with the results of the present study, which display a significant association between iodine deficiency and an elevated risk of hyperglycemia. These studies provide a more comprehensive biological insight into the adverse effects of iodine deficiency on human health. Further stratified analysis indicates that the association between iodine deficiency and hyperglycemia was observed exclusively in males, whereas the association with hypertension was present solely in females. Studies have found that estrogen can protect females from insulin resistance, thereby maintaining stable blood sugar levels [39]. In comparison, studies have shown that males carrying transcriptional variants of the Androgen Receptor (AR) gene with reduced activity are more susceptible to hyperinsulinemia [40]. This observation is consistent with the present study’s finding of an association between iodine deficiency and hyperglycemia in males. Additionally, postmenopausal females exhibit a steeper age-related increase in blood pressure compared with males, probably attributable to the decline in estrogen secretion [41]. A study on elderly males found that total testosterone levels were negatively correlated with SBP and the subsequent 20-year mortality risk [42]. Furthermore, a short-term crossover study revealed that testosterone replacement therapy could lower DBP in obese males [43]. These findings collectively emphasize the clinical and public health significance of iodine status, indicating that iodine deficiency may be a modifiable risk factor for hypertension and hyperglycemia.

Moreover, iodine deficiency is significantly associated with elevated odds of hypertension and hyperglycemia in the older age group, whereas no such association is evident among the younger adults aged 18 to 50 years. Studies have shown that muscle mass begins to decline at a rate of approximately 0.5–1.0% per year starting in middle age [44]. Even among individuals who engage in lifelong physical activity or are highly trained athletes, muscle quality and physical performance gradually deteriorate with aging [45], illustrating the decline in metabolic capacity in older adults. Younger individuals exhibit greater metabolic compensatory capacity, which may mitigate the adverse effects of mild iodine deficiency. However, the body’s compensatory mechanisms gradually weaken with age, leading to the manifestation of accumulated effects of iodine deficiency. This phenomenon may explain why the association between iodine deficiency and the risks of hypertension and hyperglycemia is only observed in the older age group (above 50 years).

This study finds that vitamin A deficiency may potentiate the risk of hyperglycemia associated with urinary iodine deficiency, whereas individuals with sufficient vitamin A do not exhibit this increased risk. Vitamin A and its metabolites affect insulin sensitivity and pancreatic β-cell function [19]. Although no direct comparative studies have examined the interactions among vitamin A, urinary iodine levels, and blood glucose to date, independent studies have confirmed the roles of these two nutrients in glucose metabolism. A study demonstrated that vitamin A supplementation in pregnant females with malaria infection was associated with significantly higher blood glucose levels in their newborns (*p* < 0.05) [46]. A randomized controlled trial indicated that vitamin A supplements could improve the metabolic status of obese patients [23]. Consequently, vitamin A deficiency may impair the compensatory response to insulin resistance, thereby exacerbating metabolic disturbances associated with urinary iodine deficiency. Furthermore, this study reveals that vitamin A deficiency strengthens the association between urinary iodine deficiency and elevated blood glucose levels, with this moderating effect being more pronounced in men and older individuals. This observation aligns with prior findings, as these populations exhibit a higher baseline susceptibility to hyperglycemia associated with urinary iodine deficiency. Among these metabolically vulnerable individuals, the concurrent presence of two micronutrient deficiencies, iodine and vitamin A, may impose a double burden, substantially increasing the risk of blood glucose metabolism dysregulation [47].

The stronger associations observed among individuals with vitamin A deficiency may be explained by biological interactions in which vitamin A deficiency exacerbates the metabolic consequences of iodine deficiency. Vitamin A deficiency has been shown to impair multiple components of the pituitary–thyroid axis, including reduced efficiency of thyroid hormone synthesis, altered thyrotropin secretion, and diminished peripheral conversion of thyroxine to triiodothyronine [48,49]. Evidence from controlled studies indicates that vitamin A deficiency combined with iodine deficiency produces more pronounced reductions in circulating thyroid hormones and greater thyroidal stress than iodine deficiency alone, suggesting that inadequate retinoid availability amplifies the endocrine consequences of iodine depletion [49,50]. Thyroid hormones play central roles in lipid metabolism, glucose regulation, vascular function, and antioxidant balance [51]. Therefore, impaired thyroid hormone homeostasis induced by both vitamin A deficiency and iodine deficiency may promote insulin resistance, dyslipidemia, oxidative stress, and endothelial dysfunction, which are key pathways underlying cardiometabolic disease [51,52]. This biological synergy provides a coherent explanation for the stronger associations documented in vitamin A deficient individuals and highlights the need to consider combined micronutrient insufficiency when evaluating cardiometabolic risk. In addition, it should be noted that while these stratified associations were statistically significant, it is noteworthy that the effect sizes were modest, and their clinical relevance at the individual level should be further examined in larger population cohorts.

This study offers several strengths. First, the multi-stage stratified random sampling design supports the representativeness of the study population within the region and enhances statistical power for subgroup analyses. Second, clinical and laboratory measurements were obtained using standardized protocols and high-quality assays (including LC-MS/MS for vitamin quantification), and we adjusted for a range of plausible confounders while explicitly testing effect modification by vitamin A status. Third, this is the first study to use vitamin A as a stratification variable when examining the associations between urinary iodine deficiency and blood pressure, blood glucose, and blood lipids among adults. Therefore, our study provides novel epidemiological evidence suggesting that vitamin A status may modify the association between iodine deficiency and specific cardiometabolic risk factors. This represents an incremental step beyond the established links between iodine deficiency and metabolic health.

Several limitations should also be considered. First, the cross-sectional design of this study prevents the determination of causal relationships between iodine status, vitamin A, and cardiometabolic risk factors. Reverse causation cannot be excluded, and further prospective studies are thus warranted to verify the findings. Second, we relied on biochemical markers (spot UIC and serum retinol) rather than dietary intake assessments. While these markers can better reflect recent nutritional status according to the WHO guidelines, they do not capture long-term dietary patterns or total intake from food sources. For instance, iodine status was assessed from a single spot urinary iodine concentration, which reflects short-term intake and may introduce exposure misclassification. Third, approximately 30% of participants lacked validated vitamin A measurements, which could affect precision and introduce selection bias in stratified analyses. Although those with missing data were slightly younger, the differences in the distributions of key demographic and clinical characteristics between the included and original participants were mostly less than 5%. This suggests that while the potential for selection bias cannot be excluded, its magnitude is likely limited and unlikely to have substantially altered the effect estimates. Meanwhile, our stratified analyses resulted in smaller sample sizes within specific subgroups. These findings should be interpreted with caution, as reduced statistical power may affect the precision of estimates, and chance associations cannot be fully excluded. Fourth, the subgroup of participants with vitamin A deficiency was substantially smaller than the group with sufficient vitamin A. This imbalance, coupled with the missing data, implied that the observed associations within the vitamin A deficiency subgroup should be interpreted with particular caution, as they might be less stable and generalizable. The results warrant confirmation in larger studies with a more balanced distribution of vitamin A status. Finally, residual confounding from unmeasured dietary factors, medication use, or other lifestyle variables cannot be fully excluded. Meanwhile, the study participants were recruited from a single province in China. The regional specificity of the sample may limit the generalizability of our findings to other populations and warrants external validation.

## 5. Conclusions

In this cross-sectional study, we found that iodine deficiency was associated with adverse levels of blood pressure, fasting glucose, and total cholesterol. Our analyses suggest that these associations may be modified by vitamin A status, potentially being stronger among individuals with concurrent subclinical vitamin A deficiency, particularly for fasting blood glucose and total cholesterol. These findings require confirmation in studies with a more balanced micronutrient status distribution. Future prospective and intervention studies are thus needed to confirm these findings and to evaluate if combined micronutrient supplementation can reduce cardiometabolic risk.

## Figures and Tables

**Table 1:** Basic demographic information of the participants.

Variables	Total (*n* = 4723)	No Vitamin A Data (*n* = 1459)	Vitamin A Sufficient (*n* = 554)	Vitamin A Deficiency (*n* = 2710)	*p*-Value
Height (cm, mean ± SD)	162.05 ± 8.37	160.42 ± 8.10	165.39 ± 8.16	162.24 ± 8.33	<0.001
Weight (kg, mean ± SD)	62.64 ± 11.32	61.84 ± 11.08	67.24 ± 10.62	62.13 ± 11.36	<0.001
BMI (kg/m^2^, mean ± SD)	23.80 ± 3.61	23.98 ± 3.55	24.53 ± 3.07	23.55 ± 3.72	<0.001
Sex (*n* (%))					
Male	1895 (40.12)	549 (37.63)	392 (70.76)	954 (35.20)	<0.001
Female	2828 (59.88)	910 (62.37)	162 (29.24)	1756 (64.80)	
Age group (*n* (%))					
18–50 years old	2174 (46.03)	560 (38.38)	188 (33.94)	1426 (52.62)	<0.001
>50 years old	2549 (53.97)	899 (61.62)	366 (66.06)	1284 (47.38)	
SBP (mmHg)	126.04 ± 21.97	130.97 ± 20.26	129.81 ± 15.24	122.62 ± 23.34	<0.001
DBP (mmHg)	77.95 ± 17.43	80.14 ± 11.38	80.60 ± 10.05	76.24 ± 20.79	<0.001
FBG (mmol/L)	5.28 ± 1.61	5.31 ± 1.78	5.70 ± 2.05	5.17 ± 1.39	<0.001
TC (mmol/L)	4.91 ± 1.00	5.03 ± 1.05	5.15 ± 1.02	4.81 ± 0.95	<0.001
HDL-C (mmol/L)	1.34 ± 0.35	1.33 ± 0.35	1.26 ± 0.34	1.37 ± 0.35	<0.001
LDL-C (mmol/L)	2.81 ± 0.84	2.82 ± 0.89	2.87 ± 0.92	2.80 ± 0.80	0.075
TGs (mmol/L)	1.71 ± 1.52	1.81 ± 1.87	2.50 ± 1.95	1.49 ± 1.09	<0.001
Hypertension					
No	3630 (76.87)	944 (64.70)	394 (71.25)	2292 (84.58)	<0.001
Yes	1092 (23.13)	515 (35.30)	159 (28.75)	418 (15.42)	
Hyperglycemia					
No	3818 (85.57)	1208 (82.91)	432 (79.85)	2178 (88.39)	<0.001
Yes	644 (14.43)	249 (17.09)	109 (20.15)	286 (11.61)	
High TC					
No	4275 (90.53)	1291 (88.55)	479 (86.46)	2505 (92.44)	<0.001
Yes	447 (9.47)	167 (11.45)	75 (13.54)	205 (7.56)	
Low HDL-C					
No	3830 (81.11)	1180 (80.93)	405 (73.10)	2245 (82.84)	<0.001
Yes	892 (18.89)	278 (19.07)	149 (26.90)	465 (17.16)	
High LDL-C					
No	4423 (93.67)	1362 (93.42)	508 (91.70)	2553 (94.21)	0.033
Yes	299 (6.33)	96 (6.58)	46 (8.30)	157 (5.79)	
High TGs					
No	3770 (79.84)	1145 (78.53)	319 (57.58)	2306 (85.09)	<0.001
Yes	952 (20.16)	313 (21.47)	235 (42.42)	404 (14.91)	
Smoking status (*n* (%))					
Smoke every day	635 (13.44)	193 (13.23)	156 (28.16)	286 (10.55)	<0.001
Smoke occasionally	82 (1.74)	27 (1.85)	15 (2.71)	40 (1.48)	
Do not smoke now	258 (5.46)	63 (4.32)	55 (9.93)	140 (5.17)	
Never smoke	3521 (74.55)	1175 (80.53)	320 (57.76)	2026 (74.76)	
Missing	227 (4.81)	1 (0.07)	8 (1.44)	218 (8.04)	
Dietary habits					
Frequently consume seafood	1004 (21.26)	260 (17.82)	136 (24.55)	608 (22.44)	<0.001
Frequently eat pickled foods	179 (3.79)	46 (3.15)	31 (5.60)	102 (3.76)	
Frequently drink vegetable soup	387 (8.19)	189 (12.95)	42 (7.58)	156 (5.76)	
Others	2702 (57.21)	596 (40.85)	344 (62.09)	1762 (65.02)	
Missing	451 (9.55)	368 (25.22)	1 (0.18)	82 (3.03)	
Sleeping time					
≥8 h	3170 (67.12)	756 (51.82)	395 (71.30)	2019 (74.50)	<0.001
<8 h	1092 (23.12)	330 (22.62)	156 (28.16)	606 (22.36)	
Missing	461 (9.76)	373 (25.57)	3 (0.54)	85 (3.14)	
Vitamin D levels (*n* (%))					
Sufficient	1166 (25.10)	463 (33.36)	188 (34.06)	515 (19.03)	<0.001
Deficiency	3480 (74.90)	925 (66.64)	364 (65.94)	2191 (80.97)	

Note: SBP, systolic blood pressure; DBP, diastolic blood pressure; FBG, fasting blood glucose; TC, total cholesterol; HDL-C, high-density lipoprotein cholesterol; LDL-C, elevated low-density lipoprotein cholesterol; TGs, triglycerides. *p*-values, the statistical differences in the distributions between vitamin A sufficient and vitamin A deficiency groups.

**Table 2:** Association between iodine deficiency and blood pressure, blood glucose, and blood lipid levels.

Outcome	Adjusted Model	
	*β* (95%CI)	*p* Value
SBP	2.89 (2.00–3.77)	<0.001
DBP	1.08 (0.55–1.60)	<0.001
FBG	0.06 (0.01–0.12)	0.031
TC	0.05 (0.00–0.10)	0.033
HDL-C	0.00 (−0.02–0.01)	0.738
LDL-C	0.04 (−0.01–0.08)	0.099
TGs	0.00 (−0.06–0.05)	0.882
	**OR (95% CI)**	***p*** **Value**
Hypertension	1.41 (1.23–1.63)	<0.001
Hyperglycemia	1.39 (1.17–1.65)	<0.001
High TC	1.09 (0.89–1.33)	0.404
Low HDL-C	0.95 (0.81–1.11)	0.521
High LDL-C	1.06 (0.83–1.35)	0.652
High TGs	1.03 (0.89–1.20)	0.665

Note: Results were presented as numerical changes for continuous outcomes and odds ratios (ORs) for binary outcomes and their 95% confidence intervals (CIs). Age, gender, BMI, smoking status, dietary habits, sleeping time, and vitamin D levels were adjusted. SBP, systolic blood pressure; DBP, diastolic blood pressure; FBG, fasting blood glucose; TC, total cholesterol; HDL-C, high-density lipoprotein cholesterol; LDL-C, elevated low-density lipoprotein cholesterol; TGs, triglycerides.

## Data Availability

The data are not publicly available due to privacy. The raw data supporting the conclusions of this article will be made available upon request.

## Supplementary Materials

The following supporting information can be downloaded at: https://www.mdpi.com/article/10.3390/nu17243948/s1, Table S1: Basic demographic information of the participants with available vitamin A data; Table S2: Association between iodine deficiency and blood pressure, blood glucose, and blood lipid levels, stratified by gender; Table S3: Association between iodine deficiency and blood pressure, blood glucose, and blood lipid levels, stratified by age group; Table S4: Association between iodine deficiency and blood pressure, blood glucose, and blood lipid levels, stratified by Vitamin A and gender group; Table S5: Association between iodine deficiency and blood pressure, blood glucose, and blood lipid levels, stratified by Vitamin A and age group; Table S6: Association between iodine deficiency and blood pressure, blood glucose, and blood lipid levels, stratified by the median vitamin A value of the participants.
