# Supplementary material for: Effects of Iodine Status and Vitamin A Level on Blood Pressure, Blood Glucose, and Blood Lipid Levels in Chinese Adults: A Cross-Sectional Study
Source: Nutrients. 2025 Dec 17;17(24):3948. doi: 10.3390/nu17243948 (PMC12735855; doi:10.3390/nu17243948)
Supplement: Supplementary file 1 [file nutrients-17-03948-s001.zip › nutrients-3991918-supplementary.pdf]

Supplementary materials

Table S1. Basic demographic information of the participants with available vitamin A data.

Variables	Total (N=4723)	Participants with available vitamin A data (N=3264)
Height (cm, mean ± SD)	162.05±8.37	162.77±8.39
Weight (kg, mean ± SD)	62.64±11.32	63.00±11.40
BMI (kg/m^2, mean ± SD)	23.80±3.61	23.72±3.64
Sex (N (%))		
Male	1895(40.12)	1346(41.24)
Female	2828(59.88)	1918(58.76)
Age group (N (%))		
18-50 years old	2174(46.03)	1614(49.45)
> 50 years old	2549(53.97)	1650(50.55)
SBP (mmHg)	126.04±21.97	123.84±22.34
DBP (mmHg)	77.95±17.43	76.98±19.46
FBG (mmol/L)	5.28±1.61	5.26±1.53
TC (mmol/L)	4.91±1.00	4.87±0.97
HDL-C (mmol/L)	1.34±0.35	1.35±0.35
LDL-C (mmol/L)	2.81±0.84	2.81±0.82
TGs (mmol/L)	1.71±1.52	1.66±1.33
Hypertension		
No	3630(76.87)	2686(82.32)
Yes	1092(23.13)	577(17.68)
Hyperglycemia		
No	3818(85.57)	2610(86.86)
Yes	644(14.43)	395(13.14)
High TC		
No	4275(90.53)	2984(91.42)
Yes	447(9.47)	280(8.58)
Low HDL-C		
No	3830(81.11)	2650(81.19)
Yes	892(18.89)	614(18.81)
High LDL-C		
No	4423(93.67)	3061(93.78)
Yes	299(6.33)	203(6.22)
High TGs		
No	3770(79.84)	2625(80.42)
Yes	952(20.16)	639(19.58)
Smoking status (N (%))		
Smoke every day	635(13.44)	442(13.54)
Smoke occasionally	82(1.74)	55(1.69)
Don't smoke now	258(5.46)	195(5.97)
Never smoke	3521(74.55)	2346(71.88)
Missing	227(4.81)	226(6.92)
Dietary habits		
Frequently consume seafood	1004(21.26)	744(22.79)
Frequently eat pickled foods	179(3.79)	133(4.07)
Frequently drink vegetable soup	387(8.19)	198(6.07)
Others	2702(57.21)	2106(64.52)
Missing	451(9.55)	83(2.54)
Sleeping time		
≥8h	3170(67.12)	2414(73.96)
＜8h	1092(23.12)	762(23.35)
Missing	461(9.76)	88(2.70)
Vitamin D levels (N (%))		
Sufficient	1166(25.10)	703(21.58)
Deficiency	3480(74.90)	2555(78.42)

Note: SBP, systolic blood pressure; DBP, diastolic blood pressure; FBG, fasting blood glucose; TC, total cholesterol; HDL-C, high-density lipoprotein cholesterol; LDL-C, elevated low-density lipoprotein cholesterol; TGs, triglycerides.

Table S2. Association between iodine deficiency and blood pressure, blood glucose, and blood lipid levels, stratified by gender.

Outcome	Male		Female	
	β (95%CI)	P value	β (95%CI)	P value
SBP	1.82 (0.28-3.36)	0.021	4.48 (2.64-6.33)	<0.001
DBP	-0.12 (-1.14-0.89)	0.811	1.33 (-0.22-2.88)	0.093
FBG	0.26 (0.09-0.44)	0.003	0.07 (-0.03-0.18)	0.169
TC	0.02 (-0.07-0.11)	0.634	0.08 (0.01-0.16)	0.034
HDL-C	0.01 (-0.02-0.04)	0.711	-0.01 (-0.03-0.02)	0.579
LDL-C	0.01 (-0.07-0.09)	0.784	0.04 (-0.02-0.10)	0.228
TGs	-0.11 (-0.28-0.06)	0.213	0.07 (-0.02-0.17)	0.139
	OR (95% CI)	P value	OR (95% CI)	P value
Hypertension	1.07 (0.86-1.33)	0.558	1.73 (1.44-2.09)	<0.001
Hyperglycemia	1.59 (1.23-2.06)	<0.001	1.22 (0.97-1.54)	0.087
High TC	0.91 (0.63-1.31)	0.603	1.19 (0.93-1.52)	0.160
Low HDL-C	0.92 (0.74-1.14)	0.445	0.98 (0.78-1.23)	0.873
High LDL-C	0.94 (0.61-1.45)	0.782	1.13 (0.84-1.51)	0.431
High TGs	0.90 (0.72-1.13)	0.381	1.16 (0.95-1.41)	0.158

Note: Results are presented as numerical changes for continuous outcomes and odds ratios (ORs) for binary outcomes and their 95% confidence intervals (CIs). Adjusted model was adjusted for age, BMI, smoking status, dietary habits, sleeping time, and vitamin D levels. SBP, systolic blood pressure; DBP, diastolic blood pressure; FBG, fasting blood glucose; TC, total cholesterol; HDL-C, high-density lipoprotein cholesterol; LDL-C, elevated low-density lipoprotein cholesterol; TGs, triglycerides.

Table S3. Association between iodine deficiency and blood pressure, blood glucose, and blood lipid levels, stratified by age group.

Outcome	18-50 years old		>50 years old	
	β (95%CI)	P value	β (95%CI)	P value
SBP	0.27 (-1.88-2.41)	0.808	3.25 (1.87-4.62)	<0.001
DBP	-0.30 (-2.38-1.77)	0.776	0.77 (-0.02-1.56)	0.055
FBG	0.09 (-0.03-0.22)	0.146	0.12 (-0.02-0.25)	0.091
TC	0.07 (-0.02-0.16)	0.123	0.00 (-0.08-0.08)	0.975
HDL-C	0.01 (-0.02-0.04)	0.472	-0.01 (-0.03-0.02)	0.588
LDL-C	0.02 (-0.05-0.10)	0.536	0.00 (-0.07-0.07)	0.932
TGs	0.01 (-0.12-0.15)	0.841	-0.06 (-0.17-0.06)	0.331
	OR (95% CI)	P value	OR (95% CI)	P value
Hypertension	1.00 (0.73-1.35)	0.977	1.33 (1.13-1.58)	<0.001
Hyperglycemia	1.33 (0.90-1.96)	0.149	1.23 (1.01-1.49)	0.043
High TC	1.09 (0.78-1.53)	0.608	0.99 (0.77-1.28)	0.962
Low HDL-C	1.00 (0.78-1.28)	0.996	0.92 (0.75-1.12)	0.395
High LDL-C	0.96 (0.63-1.48)	0.867	1.00 (0.74-1.34)	0.991
High TGs	1.20 (0.94-1.52)	0.138	0.88 (0.73-1.07)	0.206

Note: Results are presented as numerical changes for continuous outcomes and odds ratios (ORs) for binary outcomes and their 95% confidence intervals (CIs). Adjusted model was adjusted for gender, BMI, smoking status, dietary habits, sleeping time, and vitamin D levels. SBP, systolic blood pressure; DBP, diastolic blood pressure; FBG, fasting blood glucose; TC, total cholesterol; HDL-C, high-density lipoprotein cholesterol; LDL-C, elevated low-density lipoprotein cholesterol; TGs, triglycerides.

Table S4. Association between iodine deficiency and blood pressure, blood glucose, and blood lipid levels, stratified by Vitamin A and gender group.

Outcome	Vitamin A sufficient		Vitamin A deficiency	
	β (95%CI)	P value	β (95%CI)	P value
**Male**				
SBP	0.11 (-0.37-0.60)	0.647	0.26 (0.04-0.47)	0.019
DBP	1.94 (-1.19-5.06)	0.225	-0.01 (-2.05-2.03)	0.990
FBG	-0.17 (-2.36-2.02)	0.879	-1.10 (-2.50-0.30)	0.123
TC	0.04 (-0.16-0.25)	0.682	0.11 (-0.01-0.23)	0.082
HDL-C	-0.01 (-0.08-0.06)	0.819	-0.01 (-0.05-0.03)	0.642
LDL-C	0.04 (-0.14-0.22)	0.675	0.06 (-0.04-0.17)	0.258
TGs	-0.18 (-0.64-0.29)	0.456	0.11 (-0.04-0.26)	0.148
	OR (95% CI)	P value	OR (95% CI)	P value
Hypertension	0.98 (0.60-1.60)	0.926	0.77 (0.54-1.10)	0.155
Hyperglycemia	0.85 (0.47-1.53)	0.581	1.49 (0.99-2.26)	0.058
High TC	1.13 (0.59-2.20)	0.708	1.17 (0.59-2.34)	0.647
Low HDL-C	1.13 (0.69-1.86)	0.629	0.94 (0.68-1.28)	0.679
High LDL-C	1.03 (0.44-2.41)	0.940	1.15 (0.58-2.29)	0.691
High TGs	1.06 (0.68-1.65)	0.793	1.02 (0.71-1.47)	0.910
**Female**				
	β (95%CI)	P value	β (95%CI)	P value
SBP	0.23 (-0.25-0.71)	0.350	0.08 (-0.04-0.20)	0.206
DBP	3.52 (-1.77-8.81)	0.194	1.12 (-1.48-3.73)	0.398
FBG	0.59 (-2.58-3.76)	0.717	-0.16 (-2.60-2.27)	0.897
TC	-0.14 (-0.53-0.24)	0.465	0.06 (-0.03-0.16)	0.194
HDL-C	0.01 (-0.10-0.11)	0.905	0.00 (-0.03-0.03)	0.967
LDL-C	-0.10 (-0.45-0.24)	0.562	0.06 (-0.02-0.14)	0.113
TGs	-0.22 (-0.67-0.23)	0.340	-0.04 (-0.14-0.07)	0.492
	OR (95% CI)	P value	OR (95% CI)	P value
Hypertension	1.66 (0.82-3.37)	0.159	1.23 (0.91-1.65)	0.175
Hyperglycemia	1.75 (0.84-3.68)	0.137	1.25 (0.89-1.76)	0.191
High TC	1.72 (0.74-4.03)	0.211	1.03 (0.74-1.45)	0.844
Low HDL-C	0.75 (0.33-1.68)	0.481	0.99 (0.72-1.38)	0.976
High LDL-C	1.17 (0.42-3.21)	0.765	1.28 (0.87-1.89)	0.207
High TGs	0.47 (0.23-0.94)	0.032	1.00 (0.75-1.35)	0.973

Note: Results are presented as numerical changes for continuous outcomes and odds ratios (ORs) for binary outcomes and their 95% confidence intervals (CIs). Adjusted model was adjusted for age, BMI, smoking status, dietary habits, sleeping time, and vitamin D levels. SBP, systolic blood pressure; DBP, diastolic blood pressure; FBG, fasting blood glucose; TC, total cholesterol; HDL-C, high-density lipoprotein cholesterol; LDL-C, elevated low-density lipoprotein cholesterol; TGs, triglycerides.

Table S5. Association between iodine deficiency and blood pressure, blood glucose, and blood lipid levels, stratified by Vitamin A and age group.

Outcome	Vitamin A sufficient		Vitamin A deficiency	
	β (95%CI)	P value	β (95%CI)	P value
18-50 years old				
SBP	0.29 (-0.35-0.93)	0.375	0.02 (-0.11-0.15)	0.744
DBP	-1.52 (-6.32-3.27)	0.534	-1.23 (-4.30-1.85)	0.435
FBG	-1.32 (-4.92-2.28)	0.472	-1.48 (-4.57-1.62)	0.349
TC	-0.06 (-0.38-0.26)	0.710	0.07 (-0.03-0.18)	0.161
HDL-C	-0.04 (-0.14-0.05)	0.366	0.02 (-0.02-0.05)	0.329
LDL-C	-0.15 (-0.40-0.11)	0.266	0.04 (-0.04-0.13)	0.335
TGs	0.05 (-0.67-0.77)	0.896	0.02 (-0.10-0.14)	0.744
	OR (95% CI)	P value	OR (95% CI)	P value
Hypertension	0.64 (0.27-1.50)	0.303	0.69 (0.42-1.14)	0.149
Hyperglycemia	0.64 (0.19-2.13)	0.469	1.29 (0.71-2.35)	0.409
High TC	1.01 (0.39-2.59)	0.984	1.06 (0.67-1.69)	0.800
Low HDL-C	1.28 (0.60-2.73)	0.526	0.91 (0.64-1.28)	0.588
High LDL-C	0.60 (0.15-2.48)	0.484	0.99 (0.58-1.70)	0.976
High TGs	1.71 (0.86-3.38)	0.125	1.06 (0.75-1.49)	0.742
>50 years old				
	β (95%CI)	P value	β (95%CI)	P value
SBP	0.00 (-0.45-0.46)	0.996	0.20 (0.02-0.38)	0.031
DBP	2.83 (-0.42-6.07)	0.089	0.57 (-1.20-2.34)	0.527
FBG	0.37 (-1.70-2.44)	0.726	-0.24 (-1.30-0.82)	0.656
TC	0.02 (-0.20-0.25)	0.843	0.04 (-0.07-0.14)	0.506
HDL-C	0.01 (-0.06-0.09)	0.696	-0.02 (-0.06-0.02)	0.315
LDL-C	0.06 (-0.15-0.27)	0.587	0.05 (-0.04-0.14)	0.271
TGs	-0.26 (-0.65-0.13)	0.190	-0.04 (-0.16-0.09)	0.566
	OR (95% CI)	P value	OR (95% CI)	P value
Hypertension	1.25 (0.78-2.00)	0.360	0.97 (0.74-1.26)	0.801
Hyperglycemia	1.08 (0.65-1.82)	0.758	1.23 (0.91-1.66)	0.175
High TC	1.42 (0.75-2.70)	0.283	0.97 (0.65-1.46)	0.894
Low HDL-C	0.92 (0.53-1.58)	0.751	0.99 (0.73-1.33)	0.942
High LDL-C	1.23 (0.58-2.61)	0.586	1.37 (0.88-2.13)	0.157
High TGs	0.64 (0.40-1.03)	0.066	0.91 (0.67-1.25)	0.568

Note: Results are presented as numerical changes for continuous outcomes and odds ratios (ORs) for binary outcomes and their 95% confidence intervals (CIs). Adjusted model was adjusted for gender, BMI, smoking status, dietary habits, sleeping time, and vitamin D levels. SBP, systolic blood pressure; DBP, diastolic blood pressure; FBG, fasting blood glucose; TC, total cholesterol; HDL-C, high-density lipoprotein cholesterol; LDL-C, elevated low-density lipoprotein cholesterol; TGs, triglycerides.

Table S6. Association between iodine deficiency and blood pressure, blood glucose, and blood lipid levels, stratified by the median vitamin A value of the participants.

Outcome	Vitamin A sufficient		Vitamin A deficiency		P for interaction
	β (95%CI)	P value	β (95%CI)	P value	
SBP	1.39 (-0.89-3.67)	0.232	0.74 (-1.23-2.72)	0.461	0.332
DBP	-0.41 (-1.90-1.07)	0.586	-0.39 (-2.20-1.42)	0.675	0.497
FBG	0.17 (-0.11-0.44)	0.240	0.13 (0.01-0.24)	0.031	0.412
TC	0.01 (-0.14-0.16)	0.864	0.07 (-0.01-0.15)	0.070	0.180
HDL-C	-0.02 (-0.07-0.03)	0.484	0.00 (-0.03-0.03)	0.875	0.121
LDL-C	0.00 (-0.13-0.13)	0.988	0.06 (-0.01-0.13)	0.079	0.286
TGs	-0.04 (-0.30-0.22)	0.764	-0.01 (-0.10-0.07)	0.758	0.184
	OR (95% CI)	P value	OR (95% CI)	P value	
Hypertension	1.10 (0.78-1.54)	0.582	0.97 (0.76-1.24)	0.834	0.224
Hyperglycemia	1.27 (0.86-1.86)	0.226	1.27 (0.96-1.69)	0.095	0.488
High TC	1.39 (0.89-2.16)	0.146	0.99 (0.71-1.37)	0.943	0.152
Low HDL-C	0.87 (0.61-1.25)	0.461	1.03 (0.81-1.30)	0.833	0.239
High LDL-C	1.11 (0.65-1.89)	0.700	1.23 (0.86-1.77)	0.255	0.320
High TGs	0.95 (0.69-1.29)	0.722	0.98 (0.76-1.25)	0.845	0.279

Note: Results are presented as numerical changes for continuous outcomes and odds ratios (ORs) for binary outcomes and their 95% confidence intervals (CIs). Adjusted model was adjusted for age, gender, BMI, smoking status, dietary habits, sleeping time, and vitamin D levels. SBP, systolic blood pressure; DBP, diastolic blood pressure; FBG, fasting blood glucose; TC, total cholesterol; HDL-C, high-density lipoprotein cholesterol; LDL-C, elevated low-density lipoprotein cholesterol; TGs, triglycerides. P for interaction, the statistical significance of the multiplicative interaction terms of iodine deficiency and vitamin A levels.
